# A method for rapid high-throughput biophysical analysis of proteins

**DOI:** 10.1038/s41598-017-08664-w

**Published:** 2017-08-22

**Authors:** Albert Perez-Riba, Laura S. Itzhaki

**Affiliations:** Department of Pharmacology University of Cambridge, Tennis Court Road, Cambridge, CB2 1PD UK

## Abstract

Quantitative determination of protein thermodynamic stability is fundamental to many research areas, both basic and applied. Although chemical-induced denaturation is the gold-standard method, it has been replaced in many settings by semi-quantitative approaches such as thermal stability measurements. The reason for this shift is that chemical denaturation experiments are labour-intensive, sample-costly and time-consuming, and it has been assumed that miniaturisation to a high-throughput format would not be possible without concomitantly comprising data quality. Here we exploit current technologies to create a high-throughput label-free chemical denaturation method that is capable of generating replicate datasets on multiple proteins in parallel on a timescale that is at least ten times faster, much more economical on sample, and with the potential for superior data quality, than the conventional methods used in most research labs currently.

## Introduction

Here we exploit the capabilities of the latest generation of commercially available plate readers combined with a small-scale protein purification procedure to produce a method for high-throughput quantitative measurement of protein stability. Chemical-induced equilibrium denaturation monitored by intrinsic protein fluorescence is the gold standard, but in many research fields such as protein engineering and design, drug discovery, and other medium- and high-throughput endeavours it is often not the method of choice^1^. Alternatives, like differential scanning calorimetry, which often only provide a semi-quantitative measure of stability, tend to be preferred because chemical denaturation generally requires large sample quantities and is time-consuming and labour-intensive. It was over ten years ago that Dalby and co-workers reported success in using a plate format to perform chemical denaturation and highlighted the tremendous potential that such a high-throughput approach would have in both academia and industry^2^. The method was also used to measure ligand-induced stability changes and, thereby, dissociation constants for ligand binding^3^. However, data quality and reproducibility were inferior to those obtained by the conventional fluorimeter method, and therefore there was no wide uptake.

Plate-reader sensitivity in the UV range has improved tremendously in recent years, and we demonstrate that it can now provide a very fast and accurate method for chemical denaturation measurements on most proteins. Moreover, it is extremely economical on sample compared with conventional fluorimeter-based methods, thereby obviating the need for the very time-consuming process of purifying proteins on a large-scale from litres of *E. coli* culture. Thus, the rapid high-throughput approach we describe here incorporates the plate-reader measurement into a protocol for small-scale protein expression and purification, enabling multiple technical and biological replicates of protein stability to be acquired in parallel on a very short timescale: 2 days from cell transformation to obtain data on 12 different proteins. All of our denaturation curves comprise at least three technical replicates (i.e. plate preparations), and each measurement is recorded ten times to further minimise the experimental error. For additional accuracy (e.g. to measure very small stability changes upon mutation), one can also carry out three biological replicates in parallel with very little additional time required. Lastly, the plate-reader approach means that we avoid the time-consuming and labour-intensive process of sample equilibration. In contrast, performing these types of replicates using the conventional fluorimeter instrumentation and the standard large-scale purification methods that are needed to generate sufficient protein for this instrumentation makes the acquisition of a single denaturation curve a labour-intensive and time-consuming task that lacks any real scope for parallelisation.

## Results

**Figure 1:**
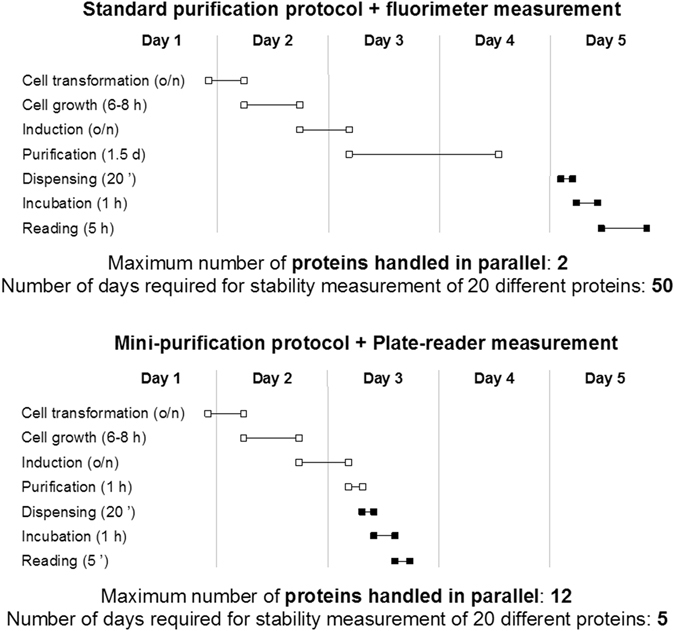
Comparison of timescales (based on an 8-hour working day) for protein production and stability measurement using our miniaturised method versus the standard method. The power of our method lies in its ability to produce data on multiple proteins in parallel, leading to an order of magnitude reduction in overall experimental time (for 20 proteins, 5 days versus 50 days). Moreover, the data produced by the miniaturised method are potentially of higher quality that those produced by the standard method, as biological replicates can be performed in parallel (i.e. with little additional experimental time) with our method.

Using our method as many as twelve different proteins can be expressed and purified in 24 hours, with a further 24 hours for stability measurement of all of them (Fig. 1). Furthermore, we have tested the method on a range of different proteins of varying stability and solubility, and we show that it is capable of yielding sufficient protein for three plates and, thereby, three technical replicates. We achieve parallel protein production by using commercially available reagents that use a combination of mild non-cationic detergents and proteases to yield very pure samples^4, 5^. The method is also suitable for the purification of proteins from inclusion bodies. Biological replicates can, of course, be obtained by making three small-scale purifications in parallel. We anticipate that our method will be very broadly applicable, and we demonstrate its scope using proteins that express at low yield and have low stability and solubility. Thus, by downscaling both the protein production and the biophysical measurement, we have a fast yet very accurate protocol for protein stability/ligand binding analysis, reducing by an order of magnitude the time taken to characterise large numbers of proteins (Fig. 1). Lastly, there is the potential for even greater throughput, as well as further reduction in the sample quantities required, via the use of commercially available robotic plate dispensers.

**Figure 2:**
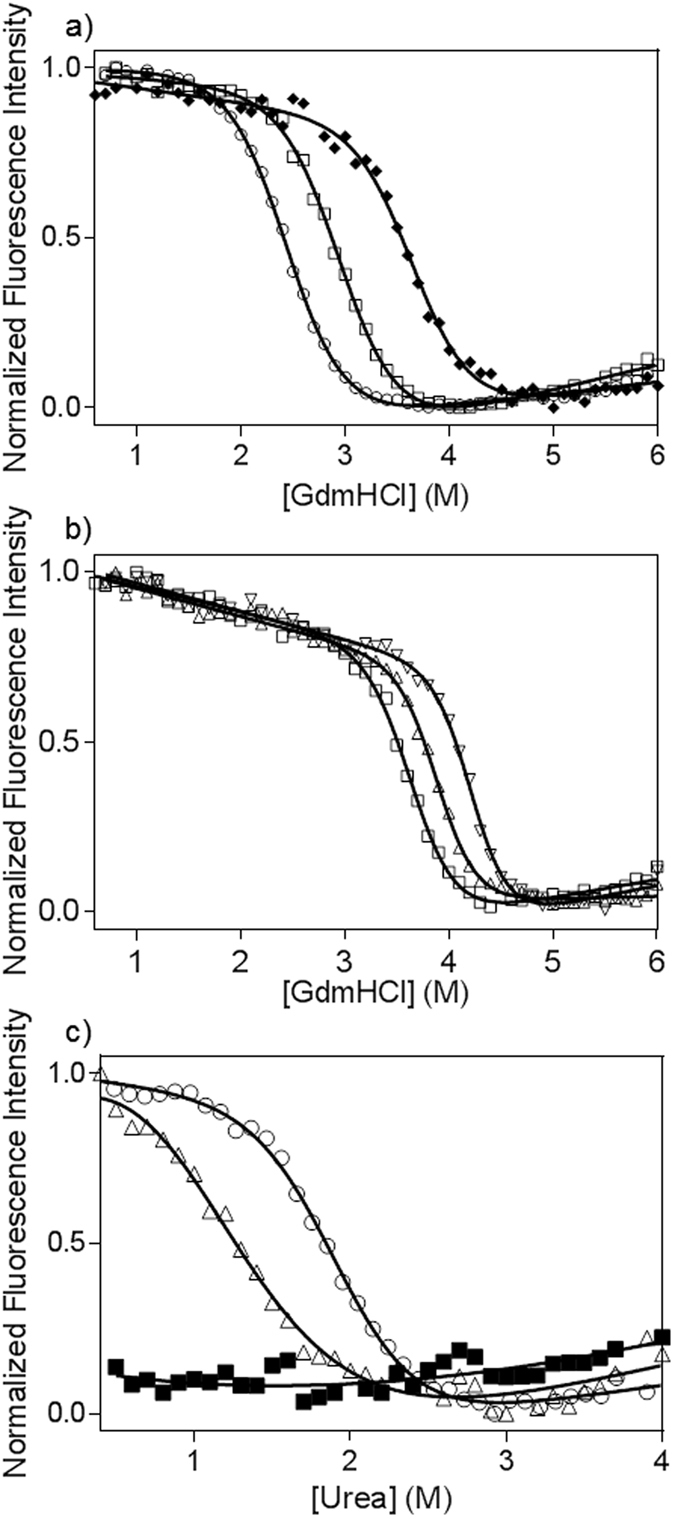
Equilibrium denaturation curves monitored by fluorescence intensity on a plate reader. The data are normalised for ease of comparison. (**a**) CTPRa (⚪), CTPRb (◻) and CTPR2c (♦). (**b**) CTPR3a (◻), CTPR4a (∆) and CTPR6a (∇). (**c**) Equilibrium denaturation curves of p16 wild type (⚪) and two cancer-associated mutants, D74N (∆) and V126N (◾). Protein concentrations were 1 µM. All experiments were performed in triplicate, with the exception of CTPR2c.

The first group of proteins we use to illustrate the method are consensus-designed tetratricopeptide repeat (CTPR) proteins^6^, which contain one tryptophan residue in each repeat giving rise to a decrease in fluorescence upon unfolding^7, 8^. Figure 2(a) shows denaturation curves for three different two-repeat variants, CTPR2a, CTPR2b and CTPR2c. CTPR2a and CTPR2b are the averages obtained from three replicate plates, in which the fluorescence of each well was read ten times and averaged. The data were fitted to a two-state model, and the resulting denaturation curves gave standard errors on the *m*-values of ~0.1 kcal mol^−1^ M^−1^. The data shown for CTPR2c are from a single plate. There is a small but significant decrease in signal-to-noise relative to the triplicate data of CTPR2a and CTPR2b, but the fitting error on the *m*-value is nevertheless still small. Thus, if the protein yield is low, one plate is sufficient to generate data of good quality. Figure 2(b) shows denaturation curves for CTPR3a, CTPR4a and CTPR6a (proteins comprising three, four and six repeats, respectively), each the average of three replicate plates. The nine plate readings were acquired in 4 hours and used less than 3 ml of 10 µM protein stock.

The method can also be applied to less well-behaved proteins and to proteins that do not show a large change in fluorescence on unfolding. An example of both these characteristics is tumour suppressor protein p16INK4a (referred to subsequently as p16). Wild-type p16 is not very stable, has low solubility and its single tryptophan is rather solvent-exposed and therefore displays only a small fluorescence change upon unfolding^9, 10^. Dozens of point mutations have been linked with tumorigenesis, and they have been shown to cause loss of function by dramatically destabilising the protein’s structure. These unstable mutants tend to be even more aggregation-prone than the wild type and are therefore very difficult to handle. Figure 2(c) shows refolding denaturation curves of wild-type p16 and two cancer-associated mutants (D74N and V126N). The proteins were first purified from inclusion bodies using BugBuster® protein extraction reagent to lyse the cells followed by affinity purification under denaturing conditions. The protein was taken directly from the inclusion body purification for the denaturation measurement. With this method we can express, purify and characterise up to 12 different proteins in just 4 hours. The denaturation data, showing that D74N is highly destabilising and V126N is so destabilising that it prevents the protein from refolding (Table 1), are in agreement with previous results obtained using standard methods^9–11^.

**Table 1:** Midpoints (D_50%_) and *m*-values for the denaturation data shown in Fig. 1, fitted to a two-state unfolding model. With the exception of CTPR2c, all experiments were performed in triplicate, and the errors listed are the standard errors of the mean. For CTPR2c the errors listed are the fitting errors on a single measurement.

Protein	D_50%_ (M)	*m* (kcal mol^−1^ M^−1^)
CTPR2b	2.47 ± 0.01	2.1 ± 0.1
CTPR2a	2.98 ± 0.03	2.12 ± 0.04
CTPR2c	3.67 ± 0.02	2.1 ± 0.1
CTPR3a	3.66 ± 0.04	2.93 ± 0.07
CPTR4a	3.90 ± 0.01	3.2 ± 0.1
CTPR6a	4.23 ± 0.02	3.3 ± 0.2
p16 wild type	1.91 ± 0.03	1.8 ± 0.1
p16 D74N	1.16 ± 0.04	1.7 ± 0.1
p16 V126N	n.d.	n.d.

To compare the equilibrium denaturation curves obtained using the plate-reader versus conventional methods, the stability of CTPR3a was additionally analysed by circular dichroism (CD) and by fluorescence using a fluorimeter. A single denaturation curve was obtained on each instrument, as is common practice for protein engineering/folding studies (Fig. S1(a)). Figure S1(b) shows the normalised data. The fluorimeter and plate-reader data overlay, and the CD data also give similar results within error (see D_50%_ and *m*-values listed in Table S1).

The final set of samples that we tested using the plate-reader method were a selection of previously published proteins kindly given to us by colleagues in Cambridge. They are representative of proteins having a range of different stabilities, folding behaviour (two/three-state) and also extinction coefficients (Figs S2 and S3). All gave high-quality data, and the free energies of unfolding obtained were in agreement with the previously published values^12–14^.

## Conclusions

The sensitivity of state-of-the-art plate readers now enables high-throughput and high-quality chemical denaturation measurements. Here we develop a protocol that combines this measurement with a rapid small-scale protein purification method, thereby streamlining the whole process and dramatically decreasing the timescale of such experiments and increasing the number of proteins that can be characterised in parallel. The specific advantages of our approach compared with the conventional large-scale protein production and fluorimeter method are: (1) the much greater number of data points that can be acquired within a given timeframe, as the plate format means that measurements are rapid and can be performed in parallel. This is key feature of our method because it means that one can increase the data quality, and so decrease the errors, without significantly increasing in the overall experiment time; (2) the ability to carry out multiple technical and/or biological replicates without much increase in experiment time; (3) the smaller sample quantities required in the plate reader obviate the need for the extremely time-consuming and labour-intensive process of large-scale protein production from litres of *E. coli* media. It also means that we can make measurements on low-stability and/or low-solubility proteins by performing refolding denaturation curves on an unfolded protein sample taken directly from the inclusion-body purification.

Our method will accelerate at least ten-fold the characterisation of protein libraries and mutant families, and we therefore anticipate that it will become standard practice across many research fields. Moreover, throughput can be increased still further using an automated plate dispenser. This would generate a constant pipeline of high-quality data several orders of magnitude more rapidly than the conventional methods employed in the protein-folding field to date, and it would also reduce the sample quantities required given that the small volumes obtained with nano-dispensers are fully compatible with current plate readers.

## Methods

### Small-scale purification of His-tagged proteins

Plasmids were transformed into *E. coli* C41 cells and plated overnight. 15 ml aliquots of 2xTY media (Roche) containing 0.1 mg/ml ampicillin were placed into 50 ml tubes. Several colonies were picked and resuspended in each 15 ml culture. For sufficient aeration it is important to only loosely tighten the lids of the 50 ml tubes. Cells were grown at 37 °C until OD600 of 0.6 and then induced with 0.5 mM IPTG overnight. Cells were pelleted at 3000 g (Eppendorf Centrifuge 5804) and then resuspended in 1 ml of BugBuster® cell lysis reagent. Alternatively, sonication in combination with lysozyme and DNAse I treatment can be used, but we have found that BugBuster® gives cleaner results. We recommend testing both methods, as their effectiveness may be protein-dependent. We found that 20 minutes’ incubation with 1 ml BugBuster® was sufficient to solubilize the cell pellets obtained from the 15 ml cultures and give a transparent lysate. Any precipiation observed in the lysis will be from the recombinant protein, due to low solubility or very high expression levels. These proteins can be purified from inclusion bodies. However, the soluble fraction may contain sufficient protein for the plate-reader measurement. The lysate was spun at 12000 g for 1 minute to pellet any insoluble protein and cell debris. The next step of the purification was as follows:

#### Transparent lysate

For use if the protein is soluble and can be purified in its native state. The supernatant was added to 100 µl bed volume of pre-washed Ni-NTA agarose beads. The following affinity purification was performed in batch, by washing the beads 4 times with 1 ml of buffer each time (alternatively, Qiagen Ni-NTA Spin Columns can be used). The first wash contained 10% BugBuster® solution and 30 mM imidazole in the chosen buffer. Here we used 50 mM sodium phosphate buffer pH 7.5, 150 mM NaCl. The three successive washes had 30 mM of imidazole in the chosen buffer. It is important to wash thoroughly to remove the detergent present in the BugBuster® solution. The detergent may be a problem for some proteins, and therefore when carried out for the first time the results should always be compared against protein purified by the standard large-scale purification method. Protein was eluted from the beads in a single step using 1 ml of chosen buffer containing 300 mM imidazole. We find that the combination of Bugbuster® and imidazole and the repeat washes in small bead volumes is sufficient to yield > 95% pure protein. Imidazole can then be removed using a NAP-5 disposable gel-filtration column (GE Healthcare).

#### Precipitate in lysate (inclusion-body purification)

The cell pellet was resuspended in 6 M UltraPure GdmHCl (MP) or 9.5 M urea. A short sonication step is advisable. The unfolded protein solution can then be added directly to 200 µl of Ni-NTA bed volume and purified using 3 washes with denaturant solution. Unlike for the transparent lysate method above, the protein is usually sufficiently pure that neither BugBuster® nor low concentrations of imidazole are needed in the wash buffer. Elution was performed in a single step using 1 ml of the denaturant solution containing 300 mM imidazole. From a 15 ml culture and for a protein of size 25 kDa, we typically obtain 1 ml of eluted protein at a concentration in the range 0.25–2.5 mg ml^−1^ (10–100 μM). The fastest procedure for the stability measurement is to dispense the eluted denatured protein into the denaturation titration (see next section). The imidazole can be removed either by dialysis or using a desalting column.

### Plate-reader measurements

Solutions of buffer and concentrated denaturant in buffer are first dispensed into Greiner Bio-One® 96-well, F-bottom, black polystyrene plates (655076) using a Microlab ML510B dispenser. We typically dispense denaturation curves in 0.1 M denaturant steps. The protein is then dispensed from a 10x concentrated stock. All plate measurements were carried out on a CLARIOstar Plate Reader (BMG Labtech) with a tryptophan detection set-up comprising two filters - an excitation filter of 295 nm and an emission filter of 360 nm - and excitation and emission bandwidths of 10 nm. The 295 nm filter is used for selective excitation of tryptophan residues. Alternatively a 280 nm filter can be used to excite both tryptophan and tyrosine residues. The high sensitivity of this state-of-the-art instrument allows readings at sub-micromolar concentrations of protein (the concentration required will, of course, depend on the number of aromatic residues in the protein and the magnitude of the fluorescence change upon unfolding).

Well volume and protein concentration are important factors for optimal signal to noise, but at the same time they will have an impact on the quantity of protein used. We find that a well volume of 150 µl is sufficient. Larger volumes will give a larger signal but will require a larger quantity of protein. We recommend a 10 µM protein stock solution, which will be diluted ten-fold to 1 µM upon dispensing. With the appropriate dispenser, the total sample volume could be further reduced to 100 µl or less.

For each protein, three sets of serial dilutions were plated consecutively. These sets can be from the same sample stock (technical replicates) or from different purifications (biological replicates). Plates were covered with a Corning® 96-well microplate aluminium sealing tape to minimise evaporation, shaken for 30 s using the CLARIOstar double-orbital shaking option, and incubated at 25 °C for 1 h. The instrument settings in the BML Labtech software were set for reading through the top orbital and “precise” rather than “rapid” measurements. Both focus height and gain were set using the fluorescence of the well having the highest fluorescence intensity. The gain adjustment was then set at 70% to prevent saturation at one or other denaturation baselines. For a good signal-to-noise ratio, we recommend to aim for a 1.5–3-fold difference in fluorescence intensity between the folded and the unfolded states.

### Data analysis

The data were analysed in GraphPad Prism 6 using the following two-state equation:

1 $${\boldsymbol{F}}=\frac{({{\boldsymbol{\alpha }}}_{{\rm{N}}}+{{\boldsymbol{\beta }}}_{{\rm{N}}}[{\boldsymbol{D}}])+({{\boldsymbol{\alpha }}}_{{\rm{D}}}+{{\boldsymbol{\beta }}}_{{\rm{D}}}[{\boldsymbol{D}}]){\bf{\exp }}(\frac{{{\boldsymbol{m}}}_{{D}-{N}}([{\boldsymbol{D}}]-\,[{{\boldsymbol{D}}}_{\mathrm{50}{ \% }}])}{{\boldsymbol{RT}}})\,}{{\bf{1}}+{\bf{e}}{\bf{x}}{\bf{p}}(\frac{{{\boldsymbol{m}}}_{{D}-{N}}([{\boldsymbol{D}}]-[{{\boldsymbol{D}}}_{\mathrm{50}{ \% }}])}{{\boldsymbol{RT}}})}\,$$

where F is the fluorescence intensity, *m*_D-N_ is the *m*-value, and *D*_50%_ is the concentration of denaturant at which the signal readout is at 50% of its total value. R is the gas constant, and T is the temperature in Kelvin. The native and denatured fluorescence may show a linear dependence on denaturant concentration, resulting in sloping native and denatured baselines, and *α*_N_ and *β*_N_ and *α*_D_ and *β*_D_ are the parameters that define this linear relationship. 10 readings were taken for each well and then averaged.

The data in Fig. S3 were globally fitted in GraphPad Prism 6 using the following three-state equation:

2 $${\boldsymbol{F}}=\tfrac{({{\boldsymbol{\alpha }}}_{{\rm{N}}}+{{\boldsymbol{\beta }}}_{{\rm{N}}}[{\boldsymbol{D}}])+{\bf{\exp }}(\tfrac{{{\boldsymbol{m}}}_{{I}-{N}}([{\boldsymbol{D}}]-[{{\boldsymbol{D}}}_{{\boldsymbol{I}}-{\boldsymbol{N}}}^{50 \% }])}{{\boldsymbol{RT}}})({{\boldsymbol{F}}}_{{\boldsymbol{I}}}+({{\boldsymbol{\alpha }}}_{{\rm{D}}}+{{\boldsymbol{\beta }}}_{{\rm{D}}}[{\boldsymbol{D}}]){\bf{\exp }}(\tfrac{{{\boldsymbol{m}}}_{{D}-{I}}([{\boldsymbol{D}}]-[{{\boldsymbol{D}}}_{{\boldsymbol{D}}-{\boldsymbol{I}}}^{50 \% }])}{{\boldsymbol{RT}}})\,}{({\bf{1}}+{\bf{\exp }}(\tfrac{{{\boldsymbol{m}}}_{{I}-{N}}([{\boldsymbol{D}}]-[{{\boldsymbol{D}}}_{{\boldsymbol{I}}-{\boldsymbol{N}}}^{50 \% }])}{{\boldsymbol{RT}}}))\,({\bf{1}}+{\bf{ex}}{\bf{p}}(\tfrac{{{\boldsymbol{m}}}_{{D}-{I}}([{\boldsymbol{D}}]-[{{\boldsymbol{D}}}_{{\boldsymbol{D}}-{\boldsymbol{I}}}^{50 \% }])}{{\boldsymbol{RT}}}))}$$

where ${{\boldsymbol{D}}}_{{\boldsymbol{I}}-{\boldsymbol{N}}}^{\mathrm{50} \% }$ and ${{\boldsymbol{D}}}_{{\boldsymbol{D}}-{\boldsymbol{I}}}^{\mathrm{50} \% }$ are the midpoints for the transitions between native and intermediate states and intermediate and denatured states, respectively, and *m*_I−N_ and *m*_D−I_ are the corresponding *m*-values. F_I_ is the fluorescence of the intermediate.

### Data availability

The data that support the findings of this study are available from the corresponding author upon reasonable request.

## Electronic supplementary material

Supplementary information
