# Supplementary material for: A method for rapid high-throughput biophysical analysis of proteins
Source: Sci Rep. 2017 Aug 22;7:9071. doi: 10.1038/s41598-017-08664-w (PMC5567296; doi:10.1038/s41598-017-08664-w)
Supplement: Supplementary file 1 — Supplementary information [file 41598_2017_8664_MOESM1_ESM.pdf]

A method for rapid high-throughput biophysical analysis of proteins

Albert Perez-Riba* and Laura S. Itzhaki*

To whom the correspondence should be addressed:

Department of Pharmacology University of Cambridge, Tennis Court Road, Cambridge CB2 1PD, UK

Email: lsi10@cam.ac.uk, ap732@cam.ac.uk

Supplementary information

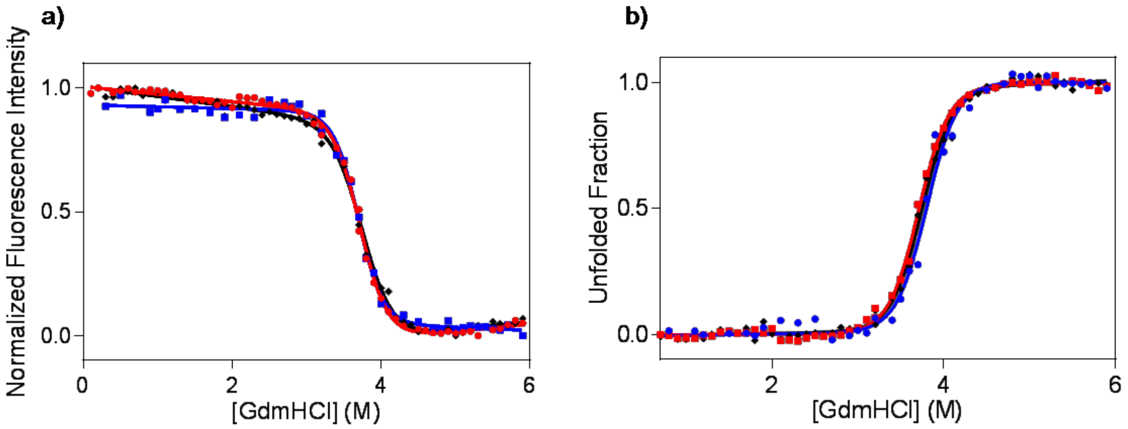

Fig. S1. Equilibrium denaturation curves of CTPR3 obtained using three different instruments. (a) Normalized signal intensity for CD (blue), fluorescence using the fluorimeter (black) and fluorescence using the plate reader (red). (b) Denaturation curves (intensity normalised relative to the unfolded protein) with baseline correction.

Table S1. $D_{50\%}$ and *m*-values obtained from a two-state fit of the data shown in Figure S1. The errors listed are the fitting errors on a single measurement.

Method	$D_{50\%}$ (M)	m (kcal mol^{-1} M^{-1})
CD	3.8 ± 0.2	4.0 ± 0.5
Fluorimeter	3.71 ± 0.01	3.2 ± 0.1
Plate reader	3.75 ± 0.01	3.1 ± 0.2

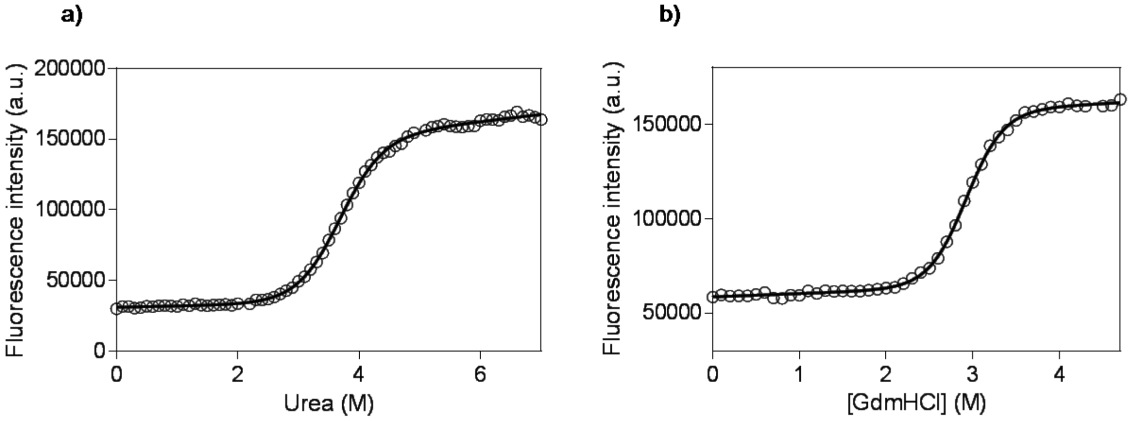

Fig S2. Equilibrium denaturation curves for spectrin and for human lysozyme mutant I59T. The conditions used are the same as those in Wensley *et. al.*[12] and Hagan *et. al.*[13].

Table S2. $D_{50\%}$ and m-values obtained from a two-state fit of the data shown in Figure S2. The errors listed are the fitting errors on a single measurement.

Protein	$D_{50\%}$ (M)	m (kcal mol^{-1} M^{-1})
R15 domain of chicken spectrin	3.70 ± 0.01	1.7 ± 0.1
Human lysozyme I59T	2.92 ± 0.01	2.7 ± 0.1

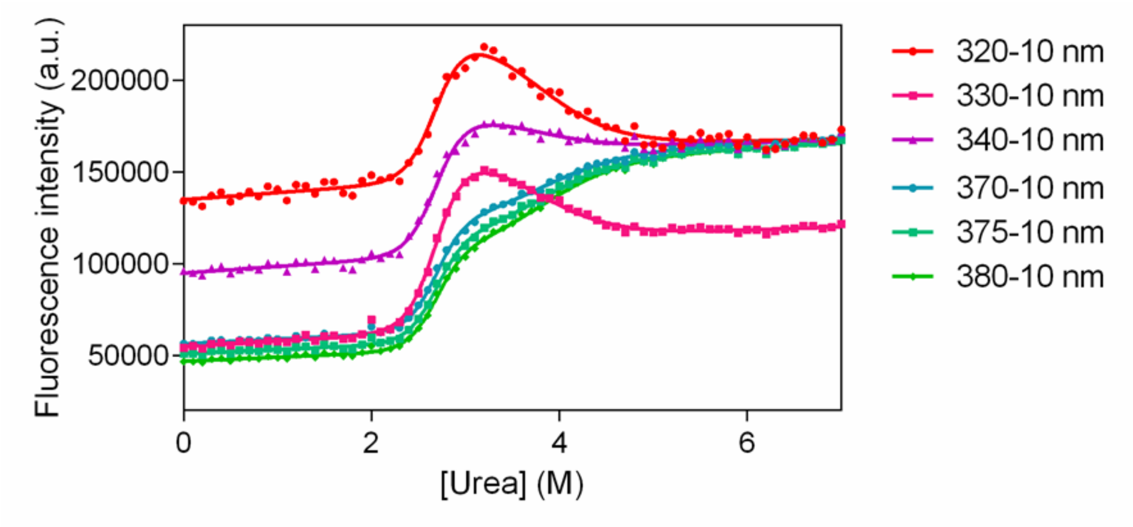

Fig S3. Equilibrium denaturation curves for UCH-L1, a knotted protein with a three-state unfolding mechanism, performed using the same conditions as Ziegler *et. al.*[14]. An emission monochromator was used, instead of using our standard emission filter (360-20 nm), to explore the full emission spectrum of the protein. The fluorescence intensity is consequently lower than that obtained with the filter, and therefore we required a protein concentration of 3 μM instead of the 1 μM we recommend in the Methods section. The six curves were globally fitted with shared two m-values and $D_{50\%}$ values (Table S3). The curves at 350-10 nm and 360-10 nm appeared two-state and therefore were not included in this analysis.

Table S3. $D_{50\%}$ and m-values obtained from a three-state fit of the data shown in Figure S3. The errors listed are the fitting errors on a single measurement.

	UCH-L1
$D_{50\%\ N\text{-}I}$ (M)	2.70 ± 0.01
$m_{\ N\text{-}I}$ (kcal mol^{-1} M^{-1})	3.7 ± 0.1
$D_{50\%\ I\text{-}D}$ (M)	3.78 ± 0.02
$m_{\ I\text{-}D}$ (kcal mol^{-1} M^{-1})	1.41 ± 0.1
